# Interprofessional Therapeutic Communication and Humanistic Practice Ability Among Saudi Nursing Students: A Cross‐Sectional Study

**DOI:** 10.1002/nop2.70906

**Published:** 2026-10-09

**Authors:** Shaherah Yousef Andargeery, Ohoud Ali Alhowaish

**Affiliations:** ^1^ Department of Nursing Management and Education, College of Nursing Princess Nourah bint Abdulrahman University Riyadh Saudi Arabia

**Keywords:** humanistic practice ability, nursing students, patient‐centred care, professional nursing education, therapeutic communication

## Abstract

**Aim:**

Humanistic practice ability refers to the capacity of nursing students to integrate ethical sensitivity, interpersonal engagement, aesthetic awareness, and compassionate, patient‐centred care into nursing practice. This study examined the levels and relationships between interprofessional therapeutic communication subscales and humanistic practice ability among undergraduate nursing students and determined the accounted variance of therapeutic communication after controlling for demographic and academic variables.

**Design:**

A cross‐sectional correlational design was used.

**Methods:**

The study was conducted with 323 second‐year undergraduate nursing students in Riyadh, Saudi Arabia. Participants completed validated self‐administered measures of interprofessional therapeutic communication and humanistic practice ability. Data were analysed using descriptive statistics, Pearson correlation, one‐way ANOVA, post hoc comparisons, and hierarchical multiple regression.

**Results:**

Students reported high levels of interprofessional therapeutic communication and humanistic practice ability. Humanistic practice ability was positively correlated with all therapeutic communication subscales except potential barriers (*r* = 0.53–0.65, *p* < 0.001). Students with advanced English proficiency demonstrated significantly higher humanistic practice ability than those with elementary and intermediate proficiency (*p* = 0.01). In the hierarchical regression model, demographic and academic variables explained 7.3% of the variance in humanistic practice ability, with English proficiency showing a high association. After adding therapeutic communication subscales, the explained variance increased significantly to 48.7% (Δ*R* ^2^ = 0.414, *p* < 0.001). Communication skills (*β* = 0.233, *p* = 0.014), patient‐centred communication (*β* = 0.253, *p* = 0.001), and English proficiency (*β* = 0.095, *p* = 0.036) were significantly associated with humanistic practice ability.

**Discussion:**

Therapeutic communication, particularly patient‐centred and relational skills, is a central determinant of perceived humanistic practice ability. Language proficiency appears to enable effective communication and humanistic care.

**Conclusion:**

Interprofessional therapeutic communication was strongly associated with humanistic practice ability among Saudi nursing students.

**Implications for Nursing:**

Curricula should integrate longitudinal, competency‐based communication training.

**Implications for Nursing Policy:**

Education standards should emphasize communication competencies and language support to strengthen patient‐centred care outcomes.

**Public Contribution:**

Nursing students participated voluntarily in the study by completing the survey. However, they were not involved in the design, conduct, reporting, or dissemination of the research.

## 1 Background

Humanistic practice ability is increasingly recognized as a foundational competency in contemporary nursing education, encompassing ethical sensitivity, interpersonal engagement, aesthetic awareness, and compassionate, patient‐centred care (Létourneau et al. 2021; Liu et al. 2023; Taghinezhad et al. 2022; Zhu et al. 2022). As healthcare becomes increasingly complex and technology driven, concerns have emerged that technical proficiency may outpace the development of humanistic competencies, potentially compromising nurse–patient relationships and holistic care (He et al. 2024; Kvande et al. 2021). Nursing education therefore must prepare graduates who are not only clinically competent but also able to practice with empathy, ethical integrity, and respect for human dignity.

Therapeutic communication is a central mechanism through which humanistic practice is expressed in nursing (Babaii et al. 2021; Mersha et al. 2023; Vega and Almeida 2023). It involves purposeful interpersonal interactions that promote trust, understanding, and collaboration among nurses, patients, and other healthcare professionals (Xue and Heffernan 2021; Younas et al. 2023). Contemporary evidence suggests that therapeutic communication extends beyond basic interpersonal skills to encompass patient‐centred dialogue, emotional attunement, ethical responsiveness, and interprofessional collaboration (Akpınar et al. 2025; Vaseghi et al. 2022; Xue and Heffernan 2021). These competencies are particularly important in culturally diverse and multilingual healthcare environments, where effective communication supports safe, respectful, and humane care.

Among undergraduate nursing students, therapeutic communication has been associated with professional identity formation, clinical confidence, ethical decision‐making, caring behaviours, and moral sensitivity (Nouri et al. 2025; Ross et al. 2024; Sayilan 2024; Su et al. 2025; Yang and Wang 2024; Yang, Li, et al. 2025). These characteristics represent important dimensions of humanistic nursing practice. However, much of the existing literature has examined communication skills and humanistic attributes separately, providing limited evidence regarding how interprofessional therapeutic communication relates to humanistic practice ability within an integrated framework.

Individual and academic characteristics may also influence students' development of communication and humanistic practice competencies. Academic performance, language proficiency, and personal responsibilities, including caring for dependents, can shape students' learning experiences, stress, and professional development (Andargeery et al. 2025; Dogham et al. 2024; Lino et al. 2022). English proficiency is particularly relevant in non‐English‐speaking countries because it may influence students' access to educational resources, communication in clinical settings, and participation in interprofessional teams (Alqahtani 2022; Peršolja 2025). Nevertheless, limited research has examined the contribution of these demographic and academic characteristics alongside therapeutic communication in explaining humanistic practice ability.

In Saudi Arabia, nursing education is undergoing substantial transformation in response to healthcare reform and Vision 2030, with increasing emphasis on quality of care, patient experience, and professional accountability. Despite these developments, empirical evidence concerning humanistic practice among Saudi undergraduate nursing students remains limited. Research in the region has predominantly focused on clinical competence, academic stress, and student satisfaction, leaving a gap in understanding the role of communication competencies in the development of humanistic nursing practice (Armah et al. 2020; Létourneau et al. 2021).

Addressing this gap may provide important evidence for strengthening nursing curricula and developing educational strategies that promote compassionate, ethical, and patient‐centred practice. Therefore, this study examined the perceived levels of and relationship between interprofessional therapeutic communication and humanistic practice ability among Saudi undergraduate nursing students. It also examined the extent to which demographic and academic characteristics and interprofessional therapeutic communication subscales accounted for variance in humanistic practice ability.

### 1.1 Research Questions

1. What are the perceived levels of and the relationship between interprofessional therapeutic communication subscales and humanistic practice ability among Saudi undergraduate nursing students?
2. To what extent do demographic and academic characteristics (age, Grade Point Average, English proficiency, caring for dependents, and nursing program choice) and interprofessional therapeutic communication subscales account for the variance in humanistic practice ability among Saudi undergraduate nursing students?

## 2 Methods

### 2.1 Study Design

A cross‐sectional, descriptive, and correlational design was employed to examine the relationships between interprofessional therapeutic communication and humanistic practice ability among undergraduate nursing students. This design was selected to allow for the simultaneous assessment of study variables and to explore the associations without manipulating the study environment.

### 2.2 Setting

The study was conducted at the College of Nursing in a major academic institution located in Riyadh, Saudi Arabia. The college offers a Bachelor of Nursing program and provides formal instruction in therapeutic communication as part of the undergraduate curriculum.

### 2.3 Sampling Design

A convenience sampling technique was used to recruit participants who met the predefined eligibility criteria. This approach was appropriate given the study's focus on students with direct exposure to formal therapeutic communication instruction. An attempted census approach was used to recruit participants from the eligible second‐year undergraduate nursing student cohort at the participating institution. All students who met the predefined eligibility criteria were invited to participate. Participation was voluntary, and students independently decided whether to complete the online survey. No individual participants were selectively recruited by the research team.

### 2.4 Participants and Sampling

Participants were eligible to participate in the study if they met the following criteria: (1) Enrollment as Bachelor of Nursing students at the participating College of Nursing in the Riyadh region, Saudi Arabia; (2) Enrollment as second‐year nursing students undertaking the Therapeutic Communication course during the study period. Students who were not enrolled in the specified course were excluded. All eligible students within the cohort were invited to participate in the study through the distributed survey invitation.

### 2.5 Sample Size

An a priori power analysis was conducted using G*Power version 3.1 to determine the minimum required sample size for multiple linear regression analysis. Assuming a medium effect size (*f* ^2^ = 0.15), a significance level of *α* = 0.05, statistical power of 95%, and three variables, the analysis indicated that a minimum sample size of 119 participants was required. The final sample exceeded this requirement, thereby ensuring adequate statistical power.

### 2.6 Measurements

#### 2.6.1 Demographic Questionnaire

A structured demographic questionnaire was used to collect information on participants' background characteristics relevant to the study objectives. The questionnaire included five items assessing age (in years), cumulative Grade Point Average, English proficiency level (categorized as elementary, intermediate, or advanced), caregiving responsibilities for dependents (yes/no), and whether enrollment in the nursing program was the participant's first choice (yes/no). These variables were selected based on their potential association with communication competencies and humanistic practice ability.

#### 2.6.2 Therapeutic Communication

Therapeutic communication was assessed using the Global International Therapeutic Communication Scale. This scale was originally developed by Campbell and Aredes (2019) and revised in 2022 (Campbell et al. 2022). The scale consists of 28 items designed to assess formal and informal patient–provider communication and to support integration into educational curricula aimed at enhancing student communication skills.

Responses are recorded on a 5‐point Likert scale ranging from 1 (Never) to 5 (Always), yielding total scores between 28 and 140. Higher scores indicate stronger therapeutic communication skills. Items 26, 27, and 28 were reverse coded prior to analysis. The scale comprises six subscales: Setting the Stage (6 items), Building Trust (3 items), Active Communication (6 items), Communication Skills (7 items), Patient‐Centred Communication (3 items), and Potential Barriers (3 items). Subscale scores were calculated by summing the relevant items, and an overall therapeutic communication score was derived by summing all items.

The scale has demonstrated strong evidence of content and construct validity in previous studies, supporting its use in educational and clinical training contexts (Campbell and Aredes 2019; Campbell et al. 2022). In the present study, internal consistency reliability was excellent for the overall scale (Cronbach's *α* = 0.946) and high across all subscales: Setting the Stage (*α* = 0.896), Building Trust (*α* = 0.822), Active Communication (*α* = 0.926), Communication Skills (*α* = 0.926), Patient‐Centred Communication (*α* = 0.872), and Potential Barriers (*α* = 0.824), indicating strong reliability of the instrument for assessing therapeutic communication among nursing students.

#### 2.6.3 Humanistic Practice Ability

Humanistic practice ability was measured using the Humanistic Practice Ability of Nursing Scale. The Humanistic Practice Ability of Nursing Scale was developed by Zhang et al. (2021). This 29‐item instrument uses a 5‐point Likert scale ranging from 1 (Not relevant) to 5 (Very relevant), with total scores ranging from 29 to 145. Higher scores reflect greater perceived humanistic practice ability.

The scale includes five subscales: Nursing Communication Ability (7 items), Psychological Adjustment Ability (4 items), Ethics and Legal Application Ability (7 items), Nursing Aesthetic Ability (4 items), and Caring Practical Ability (7 items). Subscale scores were calculated by summing responses within each domain, and the total score was obtained by aggregating all items.

The scale has demonstrated strong evidence of content and construct validity in prior research, supporting its use for assessing humanistic competencies in nursing populations (Zhang et al. 2021; Li et al. 2024). In the current study, internal consistency reliability was excellent for the overall scale (Cronbach's *α* = 0.977) and high across all subscales: Nursing Communication Ability (*α* = 0.917), Psychological Adjustment Ability (*α* = 0.886), Ethics and Legal Application Ability (*α* = 0.953), Nursing Aesthetic Ability (*α* = 0.849), and Caring Practical Ability (*α* = 0.947). These findings indicate that the scale is a reliable and psychometrically robust instrument for measuring perceived humanistic practice ability among undergraduate nursing students.

### 2.7 Pilot Study and Instrument Administration

Prior to the main data collection, a pilot study was conducted with 30 s‐year nursing students from the participating institution to assess the feasibility and comprehensibility of the study procedures and measurement instruments. The Global International Therapeutic Communication Scale and the Humanistic Practice Ability of Nursing Scale were administered in their original English versions without translation or modification. English was retained as the language of administration because it is the primary language of instruction and academic communication within the nursing program. Participants in the pilot study were asked to identify any items or instructions that were unclear or difficult to understand. No substantive concerns requiring modification of the instruments were identified. The pilot participants were not included in the final study sample.

### 2.8 Data Collection Procedures

Data were collected using a self‐administered online survey created in Microsoft Forms. Following Institutional Review Board approval, an invitation letter and survey link were distributed via academic email by the Nursing Club. The invitation letter included the study title, aim, and eligibility criteria. The survey link provided detailed information about the study purpose, number of items, estimated completion time, Institutional Review Board approval number, and contact information for the principal investigator. No written consent has been obtained from the participants as there is no participant‐identifiable data included. The data were collected between September 2026 and December 2026.

### 2.9 Ethical Considerations

Ethical approval was obtained from the IRB of Princess Nourah bint Abdulrahman University; Institutional Review Board Log Number: 25‐0444. Participation was voluntary, and participants were informed of their right to withdraw from the study at any time without penalty. Confidentiality and anonymity were assured, and no identifying information was collected. All data were stored securely and used solely for research purposes.

### 2.10 Data Analysis

Data were analysed using the Statistical Package for the Social Sciences, version 31. Descriptive and inferential statistics were employed to address the study objectives. Statistical significance was set at *p* < 0.05, unless otherwise specified. Prior to analysis, the dataset was screened for accuracy, missing values, and outliers. The proportion of missing data was minimal (< 5%), and cases with incomplete responses were handled using listwise deletion. Continuous variables were examined for normality through visual inspection of histograms, Q–Q plots, and standardized skewness and kurtosis values. All primary continuous variables demonstrated acceptable approximation to normal distribution, supporting the use of parametric tests.

Linearity and homoscedasticity were assessed using scatterplots of standardized residuals against associated values, which indicated no substantial deviations from assumptions. Multicollinearity was evaluated during regression analysis using tolerance and variance inflation factor (VIF) values, with tolerance values > 0.10 and VIF values < 5 indicating no evidence of problematic multicollinearity. Independence of errors was assessed using the Durbin–Watson statistic, which fell within acceptable limits.

Descriptive statistics, including means, standard deviations, ranges, frequencies, and percentages, were computed to summarize participants' demographic characteristics and key study variables (Table 1). These statistics were used to describe students' perceived levels of therapeutic communication and humanistic practice ability, as well as demographic and academic characteristics. For descriptive interpretation, the possible score range for each scale and subscale was divided into three equal intervals to classify scores as low, moderate, or high. The classification was based on the theoretical minimum and maximum scores for each measure.

**TABLE 1:** Demographic characteristics and descriptive statistics of study variables (*N* = 323).

Items	Range	*M* (SD)	*n* (%)
Age (years)	18–23	19.37 (0.85)	
GPA	1.98–5	3.8 (0.62)	
English proficiency level			
Elementary			36 (11.1%)
Intermediate			179 (55.40%)
Advanced			108 (33.40%)
Caring for dependents			
Yes			187 (57.90%)
No			136 (42.10%)
Nursing program was a first choice			
Yes			320 (71.20%)
No			93 (28.80%)
Therapeutic communication scale			
Setting the stage	(6–30)	20.97 (4.40)	
Building trust	(3–15)	12.50 (2.71)	
Active communication	(6–30)	26.02 (4.92)	
Communication skills	(7–35)	30.67 (5.73)	
Patient‐centred	(3–15)	12.80 (2.70)	
Potential barriers	(3–15)	8.69 (3.87)	
Overall therapeutic communication	(28–140)	115.99 (19.09)	
Humanistic practice ability scale			
Nursing communication ability	(7–35)	28.93 (5.85)	
Psychological adjustment ability	(4–20)	16.67 (3.37)	
Ethics and legal application ability	(7–35)	30.27 (5.83)	
Nursing aesthetic ability	(4–20)	16.49 (3.54)	
Caring practical ability	(7–35)	30.35 (5.77)	
Overall humanistic practice ability	(29–145)	122.71 (22.28)	

Abbreviations: %, percentage; GPA, grade point average; *M*, mean; *n*, number of participants; SD, standard deviation.

Bivariate relationships between demographic variables, therapeutic communication subscales, and humanistic practice ability were examined using Pearson product–moment correlation coefficients for continuous variables (Table 2). Pearson correlation analysis was selected after confirming linear relationships and approximate normality of variables. Correlation coefficients were interpreted based on established guidelines, with values ranging from small to large associations.

**TABLE 2:** Bivariate associations of demographic and communication variables with humanistic practice ability.

Demographics	Test	*p*
Age	*r* = −0.10	0.098
GPA	*r* = 0.17*	0.002
Caring for dependent	*F*(1, 321) = 6.53	0.084
Nursing program as a first choice	*F*(1, 321) = 0.20	0.414
Setting the stage	*r* = 0.57*	< 0.001
Building trust	*r* = 0.53*	< 0.001
Active communication	*r* = 0.63*	< 0.001
Communication skills	*r* = 0.65*	< 0.001
Patient‐centred	*r* = 0.63*	< 0.001
Potential barriers	*r* = −0.09	0.116

*Note:* *r* = Pearson correlation coefficient; *F* = *F* statistics (analysis of variance); *p*‐value = two‐tailed significance level (probability value).

* Correlation is significant at the 0.01 level (2‐tailed).

Group differences in humanistic practice ability based on categorical demographic variables (caring for dependents and nursing program as a first choice) were examined using one‐way analysis of variance. Prior to the analysis of variance, assumptions of normality and homogeneity of variances were assessed. Homogeneity of variance was evaluated using Levene's test and was satisfied in all comparisons.

To examine differences in humanistic practice ability across English proficiency levels (elementary, intermediate, and advanced), post hoc pairwise comparisons were conducted following a significant omnibus test. Mean differences, standard errors, *p*‐values, and 95% confidence intervals were reported. The post hoc procedure was selected to control for Type I error across multiple comparisons. Assumptions of independence, normality within groups, and homogeneity of variances were assessed and met prior to interpretation.

Hierarchical multiple regression analysis was conducted to examine whether therapeutic communication variables accounted for variance in humanistic practice ability beyond demographic and academic factors (Table 3). In Model 1, control variables (age, Grade Point Average, English proficiency, caring for dependents, and nursing program choice) were entered simultaneously. In Model 2, therapeutic communication subscales (setting the stage, building trust, active communication, communication skills, patient‐centred communication, and communication barriers) were added. Model fit was evaluated using the coefficient of determination (*R* ^2^), adjusted *R* ^2^, change in *R* ^2^ (Δ*R* ^2^), and *F* statistics. The statistical significance of incremental variance explained by Model 2 was assessed using the *F*‐change statistic. Regression coefficients were reported as unstandardized coefficients (*B*), standard errors (SE *B*), standardized coefficients (*β*), *t* values, *p* values, and 95% confidence intervals. Regression assumptions including linearity, normality of residuals, homoscedasticity, independence of errors, and absence of multicollinearity were evaluated and adequately met. Standardized residuals fell within acceptable limits (±3), supporting the robustness of the regression model.

**TABLE 3:** Hierarchical multiple regression analysis examining the associations with humanistic practice ability (*N* = 323).

Predictor	*B*	SE *B*	*β*	*t*	*p*	95% CI for *B*
Model 1 (control variables)						
*R* ^2^ = 0.073; Adjusted *R* ^2^ = 0.058; *F*(5, 317) = 4.99; *p* < 0.001						
Age	−2.098	1.437	−0.080	−1.46	0.145	[−4.92, 0.73]
GPA	3.890	2.111	0.108	1.84	0.066	[−0.26, 8.04]
English level	5.621	2.107	0.159	2.67	0.008**	[1.48, 9.77]
Caring for dependents	−4.770	2.453	−0.106	−1.94	0.053	[−9.60, 0.06]
Nursing program was first choice	−3.363	2.678	−0.068	−1.26	0.210	[−8.63, 1.91]
Model 2 (communication variables added)						
*R* ^2^ = 0.487; Adjusted *R* ^2^ = 0.469; ΔR^2^ = 0.414; *F*(11, 311) = 26.82; *p* < 0.001						
Age	−0.470	1.105	−0.018	−0.43	0.671	[−2.64, 1.70]
GPA	1.092	1.608	0.030	0.68	0.497	[−2.07, 4.26]
English level	3.362	1.596	0.095	2.11	0.036*	[0.22, 6.50]
Caring for dependents	−2.468	1.858	−0.055	−1.33	0.185	[−6.12, 1.19]
Nursing program was first choice	−1.762	2.040	−0.036	−0.86	0.388	[−5.78, 2.25]
Setting the stage	0.560	0.338	0.111	1.66	0.099	[−0.11, 1.23]
Building trust	−0.177	0.561	−0.022	−0.32	0.752	[−1.28, 0.93]
Active communication	0.660	0.410	0.146	1.61	0.109	[−0.15, 1.47]
Communication skills	0.906	0.367	0.233	2.47	0.014*	[0.19, 1.63]
Patient‐centred	2.088	0.626	0.253	3.34	0.001**	[0.86, 3.32]
Communication barriers	0.085	0.246	0.015	0.35	0.729	[−0.40, 0.57]

*Note:* *R* ^2^ = coefficient of determination; Adjusted *R* ^2^ = adjusted coefficient of determination; Δ*R* ^2^ = Change in coefficient of determination; *F* = *F* statistic (analysis of variance for regression model); *B* = Unstandardized coefficient; SE *B* = Standard error of the unstandardized regression coefficient; *β* = Standardized regression coefficient; *t* = *t* statistic; *p* = probability value; CI = confidence interval.

* *p* < 0.05.

** *p* < 0.01.

## 3 Results

### 3.1 Participant Characteristics and Descriptive Statistics

Detailed descriptive statistics are presented in Table 1. A total of 323 undergraduate nursing students participated in the study. Participants' ages ranged from 18 to 23 years, with a mean age of 19.37 years (SD = 0.85). The mean Grade Point Average was 3.80 (SD = 0.62). Regarding English proficiency, 11.1% of participants reported an elementary level, 55.4% an intermediate level, and 33.4% an advanced level. More than half of the participants reported caring responsibilities for dependents (57.9%), and the majority indicated that nursing was their first‐choice program (71.2%).

Mean scores for the Therapeutic Communication Scale demonstrated high levels across all subscales, with the exception of the potential barriers subscale, which was reported as moderate. Participants reported a mean overall therapeutic communication score of 115.99 (SD = 19.09) and a mean overall humanistic practice ability score of 122.71 (SD = 22.28). Among the therapeutic communication dimensions, communication skills (*M* = 30.67, SD = 5.73) and active communication (*M* = 26.02, SD = 4.92) had relatively higher observed mean scores, whereas potential barriers had a lower observed mean score (*M* = 8.69, SD = 3.87). The overall humanistic practice ability score was 122.71 (SD = 22.28), with nursing aesthetic ability (*M* = 30.35, SD = 5.77) and ethics and legal application ability (*M* = 30.27, SD = 5.83) showing relatively higher observed mean scores among the subscales.

### 3.2 Bivariate Associations

Bivariate analyses were conducted to examine associations between demographic variables, therapeutic communication dimensions, and humanistic practice ability (Table 2). Age was not significantly correlated with humanistic practice ability (*r* = −0.10, *p* = 0.098). Grade Point Average demonstrated a small but statistically significant positive correlation with humanistic practice ability (*r* = 0.17, *p* = 0.002). No significant group differences in humanistic practice ability were observed based on caring responsibilities for dependents, *F*(1, 321) = 6.53, *p* = 0.084, or whether nursing was the student's first‐choice program, *F*(1, 321) = 0.20, *p* = 0.414.

Post hoc comparisons were conducted to examine differences in humanistic practice ability across English proficiency levels. Students with advanced English proficiency demonstrated significantly higher humanistic practice ability scores compared with those at the elementary level (MD = −14.48, *p* = 0.002) and intermediate level (MD = −7.65, *p* = 0.012). No significant difference was observed between students with elementary and intermediate English proficiency (MD = −6.83, *p* = 0.34).

As shown in Table 2, all therapeutic communication dimensions, with the exception of potential barriers, showed moderate to strong positive correlations with humanistic practice ability. Significant positive correlations were found for setting the stage (*r* = 0.57, *p* < 0.001), building trust (*r* = 0.53, *p* < 0.001), active communication (*r* = 0.63, *p* < 0.001), communication skills (*r* = 0.65, *p* < 0.001), and patient‐centred communication (*r* = 0.63, *p* < 0.001). Potential barriers were weakly and negatively correlated with humanistic practice ability; however, this association did not reach statistical significance (*r* = −0.09, *p* = 0.116).

### 3.3 Hierarchical Multiple Regression Analysis

Hierarchical multiple regression analysis was performed to examine whether therapeutic communication variables accounted for variance in humanistic practice ability after controlling for demographic and academic factors (Table 3). In Model 1, age, Grade Point Average, English proficiency, caring for dependents, and whether nursing was the first‐choice program were entered as control variables. This model was statistically significant, *F*(5, 317) = 4.99, *p* < 0.001, explaining 7.3% of the variance in humanistic practice ability (*R* ^2^ = 0.073; adjusted *R* ^2^ = 0.058). Within this model, English proficiency emerged as a significant positive association (*β* = 0.159, *p* = 0.008), whereas age, Grade Point Average, caring for dependents, and program choice were not statistically significant variables.

In Model 2, therapeutic communication subscales (setting the stage, building trust, active communication, communication skills, patient‐centred communication, and communication barriers) were added. The full model was statistically significant, *F*(11, 311) = 26.82, *p* < 0.001, and accounted for 48.7% of the variance in humanistic practice ability (*R* ^2^ = 0.487; adjusted *R* ^2^ = 0.469). The inclusion of therapeutic communication variables resulted in a significant increase in explained variance (Δ*R* ^2^ = 0.414, *p* < 0.001).

In the final model, communication skills (*β* = 0.233, *p* = 0.014) and patient‐centred communication (*β* = 0.253, *p* = 0.001) were significantly positive variables of humanistic practice ability. English proficiency remained a significant variable (*β* = 0.095, *p* = 0.036). Age, Grade Point Average, caring for dependents, nursing program choice, setting the stage, building trust, active communication, and communication barriers were not statistically significant variables in the final model.

## 4 Discussion

This study examined the association between interprofessional therapeutic communication and perceived humanistic practice ability among undergraduate nursing students at a College of Nursing in Riyadh, Saudi Arabia. Overall, participants reported a mean therapeutic communication score of 115.99 (SD = 19.09) and a mean perceived humanistic practice ability score of 122.71 (SD = 22.28). The therapeutic communication dimensions were positively associated with perceived humanistic practice ability, with the exception of potential barriers. In the hierarchical regression analysis, demographic and academic characteristics explained 7.3% of the variance in perceived humanistic practice ability, while the addition of therapeutic communication dimensions increased the explained variance to 48.7% (Δ*R* ^2^ = 0.414, *p* < 0.001). Communication skills and patient‐centred communication were significantly associated with perceived humanistic practice ability in the final model, while English proficiency remained independently associated with the outcome. Given the cross‐sectional and self‐reported nature of the study, these findings should be interpreted as associations rather than evidence of causal relationships.

### 4.1 Therapeutic Communication and Perceived Humanistic Practice Ability

Participants reported a mean overall therapeutic communication score of 115.99 (SD = 19.09). Among the dimensions, communication skills had a mean score of 30.67 (SD = 5.73), active communication had a mean score of 26.02 (SD = 4.92), and potential barriers had a mean score of 8.69 (SD = 3.87). The overall perceived humanistic practice ability score was 122.71 (SD = 22.28), with nursing aesthetic ability (*M* = 30.35, SD = 5.77) and ethics and legal application ability (*M* = 30.27, SD = 5.83) showing relatively higher observed mean scores among the humanistic practice dimensions.

One possible explanation for these findings is that the participants were second‐year nursing students who had received formal instruction in therapeutic communication. Recent exposure to communication concepts may have increased students' familiarity with therapeutic interaction principles and their confidence in applying these skills in educational or clinical contexts. The educational emphasis on interpersonal communication may also be particularly relevant to humanistic nursing practice because several components of humanistic practice, including caring, ethical responsiveness, interpersonal engagement, and attention to patients' individual needs, are inherently relational. Thus, students who perceive themselves as more competent in communicating with patients may also perceive themselves as more capable of providing humanistic nursing care.

However, the observed scores should be interpreted cautiously. Both constructs were assessed using self‐reported measures, and students' perceptions of their communication competence may not necessarily correspond to their objectively observed communication performance. Students who generally have greater confidence in their interpersonal abilities may be inclined to rate both communication and humanistic practice more positively. This possibility of shared method variance is particularly relevant because both constructs involve closely related perceptions of interpersonal and professional competence. Therefore, the observed relationship may partly reflect students' overall perceptions of their professional capabilities rather than a direct relationship between objectively demonstrated communication behaviours and humanistic practice.

The observed pattern is broadly consistent with previous research emphasizing the relationship between communication competence, patient‐centred interaction, and humanistic nursing practice (Berglund et al. 2023; Létourneau et al. 2021; Mercan and Mersin 2025; Semerci and Savaş 2023). Nevertheless, differences in educational settings, cultural contexts, instruments, and student characteristics make direct comparisons difficult. The present findings therefore provide evidence of an association within the participating student population rather than evidence that therapeutic communication necessarily produces higher levels of humanistic practice ability.

### 4.2 Demographic and Academic Characteristics

The bivariate analyses indicated that Grade Point Average was positively associated with perceived humanistic practice ability, whereas age was not significantly associated with the outcome. Caring for dependents and nursing program choice were also not significantly associated with perceived humanistic practice ability. The association between Grade Point Average and humanistic practice ability was small and did not remain statistically significant after communication dimensions and other variables were included in the hierarchical regression model.

The absence of an association between age and perceived humanistic practice ability may partly reflect the relatively narrow age range of the participants. The sample consisted primarily of young undergraduate students, resulting in limited variability in age and consequently less opportunity to detect an age‐related association. Similarly, the lack of significant associations for caring responsibilities and nursing program choice may indicate that these characteristics were less closely related to students' perceived humanistic practice ability than communication‐related competencies within this particular sample. These findings should not be interpreted as evidence that such characteristics are irrelevant to humanistic nursing practice because other contextual or personal characteristics not examined in this study may be more important.

The initial association between Grade Point Average and perceived humanistic practice ability may reflect broader academic engagement or learning‐related characteristics. Students who perform well academically may also have greater exposure to educational activities that emphasize reflection, professional responsibility, and interpersonal competencies. However, because the association was no longer significant in the final regression model, academic performance did not explain unique variance in perceived humanistic practice ability after communication dimensions and other control variables were considered. This finding suggests that the initial bivariate relationship may overlap with other characteristics included in the regression model.

### 4.3 English Proficiency and Perceived Humanistic Practice Ability

English proficiency was significantly associated with perceived humanistic practice ability in both the bivariate and multivariable analyses. Students with advanced English proficiency reported higher perceived humanistic practice ability than those with elementary or intermediate proficiency. English proficiency also remained statistically significant after the communication dimensions were entered into the final regression model.

Several contextual explanations may account for this finding. English is the primary language of instruction and academic communication within the participating nursing program. Students with greater English proficiency may therefore experience fewer linguistic difficulties when engaging with course materials, participating in classroom discussions, communicating with instructors and peers, and completing communication‐focused learning activities. Greater proficiency may also be associated with greater confidence in expressing thoughts and responding to interpersonal situations in an educational environment where English is routinely used.

At the same time, English proficiency may represent broader educational experiences rather than language ability alone. Students with greater English proficiency may differ in prior educational exposure, opportunities to use English, academic confidence, or other characteristics that were not measured in this study. Consequently, the observed association should not be interpreted as evidence that increasing English proficiency would necessarily cause an improvement in humanistic practice ability. Rather, English proficiency was independently associated with perceived humanistic practice ability within this sample.

This finding is relevant to the Saudi nursing education context because effective communication in nursing education occurs across linguistic, educational, and cultural environments. Previous research has similarly highlighted the importance of communication and language competence in healthcare education and professional interaction (Ali and Watson 2018; Kwame 2024; Pandey et al. 2021; Seible et al. 2021). However, further research is needed to determine whether the observed association persists across different nursing institutions and regions of Saudi Arabia and whether it is also evident when communication competence is assessed through objective performance measures.

### 4.4 Association Between Therapeutic Communication and Perceived Humanistic Practice Ability

The hierarchical regression analysis demonstrated a substantial statistical association between therapeutic communication and perceived humanistic practice ability. Demographic and academic characteristics accounted for 7.3% of the variance in the first model, whereas adding the therapeutic communication dimensions increased the explained variance to 48.7% (Δ*R* ^2^ = 0.414, *p* < 0.001). Thus, therapeutic communication variables accounted for substantial additional variance in perceived humanistic practice ability beyond the demographic and academic characteristics included in the model.

One plausible explanation is the conceptual overlap between communication competence and humanistic nursing practice. Humanistic practice includes relational and interpersonal dimensions, while therapeutic communication involves listening, responding to patients' needs, establishing appropriate interactions, and communicating in a patient‐centred manner. Because these constructs share relational elements, students who perceive themselves as more capable communicators may also perceive themselves as more capable of providing humanistic care. The substantial increase in explained variance may therefore reflect the close conceptual relationship between the two constructs.

However, the magnitude of the explained variance should be interpreted with caution. Both therapeutic communication and humanistic practice ability were measured through self‐report instruments administered within the same survey. This creates the possibility of common‐method variance, whereby similar response tendencies may strengthen observed statistical relationships. In addition, the communication dimensions themselves were significantly correlated with one another, suggesting that they share considerable variance. Therefore, the regression findings should be understood as identifying statistical associations and additional explained variance rather than demonstrating that therapeutic communication causes changes in perceived humanistic practice ability.

### 4.5 Communication Skills and Patient‐Centred Communication

Among the therapeutic communication dimensions, communication skills and patient‐centred communication were significantly associated with perceived humanistic practice ability in the final regression model. Communication skills demonstrated a standardized coefficient of *β* = 0.233 (*p* = 0.014), while patient‐centred communication demonstrated *β* = 0.253 (*p* = 0.001). In contrast, setting the stage, building trust, active communication, and communication barriers did not retain statistical significance after the other variables were considered simultaneously.

The significance of communication skills and patient‐centred communication may reflect their particularly close conceptual relationship with the interpersonal components of humanistic nursing practice. Communication skills encompass the practical behaviours required to exchange information and respond appropriately during interactions, whereas patient‐centred communication emphasizes attention to patients' needs, preferences, perspectives, and experiences. These characteristics correspond closely with the relational aspects of humanistic nursing, which emphasize individualized and respectful interactions.

The fact that other communication dimensions were significantly correlated with perceived humanistic practice ability at the bivariate level but were not significant in the final regression model is also important. This pattern does not suggest that building trust or active communication are unimportant. Rather, their associations with the outcome may overlap with communication skills and patient‐centred communication. When the dimensions were considered simultaneously, communication skills and patient‐centred communication explained unique variance that was not accounted for by the other communication variables. This interpretation is consistent with the interrelated nature of therapeutic communication competencies and cautions against interpreting individual regression coefficients as evidence of isolated effects.

Previous literature has emphasized the importance of patient‐centred interaction, communication competence, and relational engagement in nursing practice (Kwame and Petrucka 2021; Taghinezhad et al. 2022). The present findings extend this literature by showing that these communication dimensions were particularly closely associated with students' perceptions of their humanistic practice ability. Nevertheless, longitudinal research and studies using observed communication performance are needed to determine whether these associations are maintained over time and across clinical contexts.

### 4.6 Implications for Nursing

The findings have important implications for undergraduate nursing education in Saudi Arabia. The significant associations between communication skills, patient‐centred communication, and perceived humanistic practice ability suggest that communication competencies warrant continued emphasis within nursing curricula. Communication training may be integrated longitudinally across undergraduate education through structured simulation, standardized‐patient encounters, reflective activities, and opportunities for interprofessional communication. Because the present study was cross‐sectional and based on self‐reported measures, these educational implications should be considered as areas for curriculum development rather than evidence that communication training directly improves humanistic practice ability.

The significant association between English proficiency and perceived humanistic practice ability also highlights the relevance of language competence within nursing education. Nursing programs may consider providing targeted academic and clinical English support to facilitate students' participation in communication‐based learning and interprofessional educational activities. Future longitudinal and intervention studies are needed to determine whether such educational approaches are associated with changes in students' perceived humanistic practice ability over time.

At the practice level, the findings support the development of continuing professional development programs that focus on patient‐centred communication and relational skills. Healthcare institutions in Saudi Arabia may leverage these results to inform orientation programs, competency frameworks, and performance evaluation systems that prioritize humanistic and communicative competencies alongside technical skills, thereby improving patient experience and quality of care.

Internationally, this study contributes to the growing body of evidence emphasizing therapeutic communication as a foundational mechanism through which humanistic nursing practice is developed. The findings reinforce the universality of communication skills and patient‐centred communication as key drivers of humanistic care, supporting their inclusion as core competencies in global nursing education standards and accreditation frameworks.

The demonstrated role of language proficiency also has broader implications for nursing education in multilingual and culturally diverse contexts worldwide. As nursing education becomes increasingly globalized, with students studying and practicing across linguistic and cultural boundaries, the integration of language‐sensitive pedagogies and culturally responsive communication training becomes essential. International nursing programs may draw on these findings to design curricula that explicitly link language competence, communication skills, and humanistic practice.

Moreover, the study provides context‐specific evidence from the Middle East, addressing a notable gap in the international nursing literature, which has historically been dominated by Western perspectives. This study provides context‐specific evidence regarding the association between interprofessional therapeutic communication and perceived humanistic practice ability among undergraduate nursing students at a College of Nursing in Riyadh, Saudi Arabia. The findings may provide a basis for comparison with nursing students in other educational and cultural contexts and may inform future multi‐institutional research.

### 4.7 Implications for Nursing and Health Policy

The findings highlight interprofessional therapeutic communication as an important educational factor associated with humanistic practice ability among undergraduate nursing students. At the nursing education level, programs should embed patient‐centred communication and core communication skills longitudinally across the curriculum rather than limiting training to a single course. High‐impact strategies include simulation with standardized patients, structured reflection and debriefing, objective assessment of communication competencies (e.g., OSCE communication stations), and interprofessional education activities that require collaborative, respectful dialogue. Because advanced English proficiency was associated with higher humanistic practice ability and remained significant in the final model, nursing programs should implement targeted language‐support pathways (e.g., clinical English modules, communication labs, and scaffolded writing/documentation support) to reduce inequities in learning outcomes and enhance students' capacity to express empathy and engage effectively with patients and teams.

From a health policy perspective, these results support incorporating measurable communication and humanistic care indicators into national nursing education standards, accreditation requirements, and competency frameworks. Policymakers and regulators can strengthen patient‐centred care goals by endorsing competency‐based communication benchmarks across undergraduate programs, funding simulation and skills‐lab infrastructure, and supporting faculty development in communication pedagogy and assessment. At the workforce level, healthcare organizations may align orientation and continuing professional development with patient‐centred communication and relational competencies, integrating these domains into performance evaluation and quality‐improvement initiatives to advance patient experience and care quality in line with national healthcare transformation priorities.

## 5 Limitations

Several limitations should be considered when interpreting the findings. Given the cross‐sectional correlational design, the observed relationships should be interpreted as associations rather than causal effects. Although hierarchical regression identified communication skills and patient‐centred communication as variables that significantly explained variance in perceived humanistic practice ability, these findings do not establish temporal or causal relationships. Longitudinal and intervention‐based studies are needed to determine whether changes in therapeutic communication are associated with subsequent changes in perceived humanistic practice ability. Longitudinal or intervention‐based studies are needed to examine causal pathways and changes over time. The reliance on self‐reported data may introduce response and social desirability biases, potentially inflating perceived levels of communication and humanistic competencies. Future research should incorporate multi‐method approaches, such as observational assessments or faculty evaluations, to strengthen measurement validity.

The study was conducted at a single College of Nursing in Riyadh and included second‐year nursing students enrolled in one academic program. Therefore, the external validity of the findings is limited, and the results should not be generalized to all nursing students in Saudi Arabia. Differences in institutional characteristics, curricula, educational practices, regional contexts, and student populations may influence therapeutic communication and perceived humanistic practice ability. Although all eligible students within the participating cohort were invited to participate, voluntary participation may also have introduced self‐selection bias. Future studies should include multiple nursing institutions across different regions of Saudi Arabia and, where feasible, use more representative sampling approaches to examine whether the observed associations are consistent across diverse educational settings. Finally, although key demographic and academic variables were included, other factors such as clinical exposure, institutional culture, emotional intelligence, and learning environment characteristics were not examined and might be associated with humanistic practice ability.

### 5.1 Recommendations for Future Research

Future research should employ longitudinal designs to examine the developmental trajectory of therapeutic communication and humanistic practice ability across different stages of nursing education. Such designs would enable assessment of causal relationships and the sustainability of communication competencies over time. Experimental and intervention‐based studies are also recommended to evaluate the effectiveness of targeted educational strategies such as simulation‐based learning, interprofessional education, reflective practice, and language support programs in enhancing therapeutic communication and humanistic practice ability.

To strengthen generalizability, future studies should include multi‐site samples across different regions and types of nursing institutions, as well as students at varying academic levels. Incorporating qualitative or mixed‐methods approaches may further enrich understanding of students' lived experiences and contextual factors influencing humanistic practice development.

Finally, future research should explore additional psychosocial and organizational variables, such as emotional intelligence, clinical learning environments, cultural competence, and faculty–student interactions, to develop comprehensive explanatory models of humanistic practice ability in nursing education.

## 6 Conclusion

Interprofessional therapeutic communication was strongly associated with perceived humanistic practice ability among undergraduate nursing students at the participating College of Nursing. Communication skills and patient‐centred communication were significantly associated with perceived humanistic practice ability and accounted for substantial additional variance beyond demographic and academic characteristics. English proficiency was also independently associated with perceived humanistic practice ability. Given the cross‐sectional design and self‐reported measures, these findings should be interpreted as associations rather than causal relationships. Further longitudinal and multi‐institutional research using objective measures of communication and clinical competence is warranted to examine these relationships across diverse nursing education contexts.

## Author Contributions

**Shaherah Yousef Andargeery:** conceptualization, methodology, software, validation, formal analysis, data curation, investigation, funding acquisition, visualization, resources, writing – review and editing, writing – original draft. **Ohoud Ali Alhowaish:** writing – original draft, resources, project administration, supervision, investigation.

## Funding

The authors extend their appreciation to Princess Nourah bint Abdulrahman University Researchers Supporting Project number (PNURSP2026R447), Princess Nourah bint Abdulrahman University, Riyadh, Saudi Arabia.

## Ethics Statement

IRB was obtained in accordance with the Declaration of Helsinki from the academic institution, Riyadh, Saudi Arabia. IRB Registration Number: HAP‐01‐R‐059; IRB Log Number: 25‐0444.

## Consent

No written consent has been obtained from the participants as there is no participant‐identifiable data included.

## Conflicts of Interest

The authors declare no conflicts of interest.

## Data Availability

The datasets generated and/or analysed during the current study are not publicly available due to data privacy but are available from the corresponding author upon reasonable request.
